# Proso Millet Cultivar Effects on Rheology of Dough and Quality Characteristics of Gluten-Free Breads

**DOI:** 10.3390/foods15101711

**Published:** 2026-05-13

**Authors:** Manjot Singh, Akinbode A. Adedeji

**Affiliations:** Department of Biosystems and Agricultural Engineering, University of Kentucky, Lexington, KY 40506, USA

**Keywords:** proso millet, gluten-free bread, dough rheology, creep–recovery, cultivar effect

## Abstract

Proso millet (*Panicum miliaceum* L.) is being increasingly used in gluten-free baking; however, the influence of cultivar-dependent functionality on gluten-free dough remains insufficiently characterized. This study systematically evaluated the impact of nine proso millet cultivars (Cope, Dawn, Sunrise, Earlybird, Huntsman, Minco, Panhandle, Plateau, and Rise) on dough rheology, bread quality, and texture stability in a gluten-free formulation. Dough viscoelasticity was characterized using small-amplitude oscillatory shear (G′, G″, tan δ) and creep–recovery (J_end_, J_nr_, J_r_/J, strain recovery, and t_90_). Breads were then evaluated for specific volume, crust and crumb color, and texture profile analysis (TPA) over 0, 2, and 5 days of storage. All doughs exhibited weak gel behavior (tan δ = 0.30–0.36) with G′ consistently exceeding G″. The waxy, low-amylose cultivar Plateau produced the stiffest dough (highest G′ and G″) and the lowest loaf specific volume (1.97 mL/g), whereas Rise and Earlybird yielded the greatest expansion (2.43–2.40 mL/g). Storage induced typical staling (increased firmness, decreased springiness, cohesiveness, and resilience) with cultivar-dependent retention of elastic attributes linked to rheological parameters. Overall, cultivar starch structure impacts dough viscoelasticity, loaf expansion, and texture evolution, highlighting cultivar selection as a practical route to improve gluten-free bread quality and shelf-life consistency.

## 1. Introduction

Millets are a group of small seeded crops in the Poaceae family that include pearl millet (*Pennisetum glaucum*), foxtail millet (*Setaria italica*), proso millet (*Panicum miliaceum*), and finger millet (*Eleusine coracana*) [1]. Their ability to grow under low-rainfall and high-temperature conditions and to tolerate drought makes millets important crops for food security in arid and semi-arid regions [1,2]. Among different millets, proso millet is of particular interest in the United States because it is the most cultivated millet type and is increasingly recognized as a functional ingredient for value-added food applications, including gluten-free baked goods [3,4].

Gluten-free product development continues to grow in relevance because bread is a widely consumed staple food, yet wheat-based bread is not suitable for all consumers [5]. Wheat flour has historically dominated breadmaking because gluten provides the viscoelastic network necessary for dough development and gas retention during proofing and baking, ultimately enabling a desirable loaf volume and crumb texture [6]. However, wheat and gluten avoidance are medically necessary for some consumers and preferred by others. Gluten-related disorders are commonly discussed across autoimmune, allergic, and non-autoimmune/non-allergic categories [7]. Celiac disease is an autoimmune disorder in which the ingestion of gluten leads to intestinal damage. The National Health and Nutrition Examination Survey (NHANES) reported that about 3 million or approximately one in 131 Americans may be affected by celiac disease [8]. In addition, non-celiac gluten sensitivity has been described as potentially affecting up to ~6% of the U.S. population, reinforcing the need for high-quality gluten-free alternatives [9,10].

A major technological limitation in gluten-free bread production is the absence of gluten, which compromises the formation of a continuous viscoelastic network capable of retaining gas during proofing and baking. As a result, gluten-free breads frequently exhibit reduced loaf volume, a weak internal structure, a dry and crumbly texture, and faster staling, which collectively reduce consumer acceptability [11]. To mitigate these quality limitations, gluten-free bread systems often depend on functional ingredients and formulation strategies that replace or mimic gluten’s structural role. Hydrocolloids are among the most widely used tools for this purpose because they can increase dough viscosity, improve gas retention, and contribute to network formation that stabilizes the developing crumb [4]. Prior studies have demonstrated that hydrocolloids and emulsifiers such as xanthan gum and Diacetyl tartaric acid esters of monoglycerides, alone or in combination, can improve loaf volume and crumb texture in gluten-free systems [12,13].

Within gluten-free formulations, genotype and cultivar selection represent critical yet often underexplored factors influencing dough rheology and final product quality [14,15]. Proso millet cultivars can differ in kernel characteristics and starch structure, particularly in amylose–amylopectin composition, which can shift pasting behavior, hydration response, and structure setting during baking [16,17]. A notable example described in this paper is Plateau, registered in 2014, which is characterized as the first waxy (almost amylose-free) proso millet cultivar, providing a clear contrast to non-waxy types and offering an opportunity to evaluate how amylose level influences functional behavior in gluten-free formulations [18].

Rheological testing provides a quantitative framework to connect cultivar composition and formulation strategy to processing behavior and baked quality [19]. Bread and dough systems exhibit viscoelastic behavior and rheological properties that are frequently treated as pivotal for quality assessment because they reflect the material’s ability to deform, retain gases, and transition into a stable structure during baking. In gluten-free bread formulations, where structure relies heavily on starch transformations and hydrocolloid-assisted network formation, rheology is especially informative because it can help explain why certain cultivars or formulations yield stronger internal structures, improved volumes, and more desirable texture outcomes [19,20]. Ingredient-driven changes in gluten-free dough rheology can translate directly into measurable changes in baked texture; for example, in gluten-free oat systems, dough viscoelastic moduli and creep behavior were strongly associated with dough hardness and final product mechanical strength [21].

Although gluten-free bread formulation has been widely studied, most work has emphasized formulation variables such as hydrocolloid type, starch source, water level, and processing conditions. In comparison, the role of cultivar-dependent functionality within a single gluten-free grain species remains less clearly understood. This is important because cultivars of the same crop can differ in starch composition, amylose–amylopectin ratio, hydration behavior, swelling capacity, and pasting properties, all of which may influence dough viscoelasticity, gas retention, crumb setting, and texture stability. For proso millet specifically, previous studies have demonstrated its potential as a gluten-free ingredient, but limited information is available on how individual cultivars behave in the same gluten-free bread formulation and how their dough rheology relates to bread volume and storage-related textural changes.

This study aimed to evaluate the rheological and baking performance of gluten-free bread formulations prepared from different proso millet cultivars and to establish relationships between rheological parameters and bread quality attributes. It was hypothesized that cultivar-dependent variations in starch composition, particularly amylose content, significantly influence dough viscoelasticity and bread structure. In a companion study [16], cultivar physicochemical, thermal, and pasting properties were characterized; the present work builds on those findings by focusing on rheology–quality relationships in gluten-free bread.

## 2. Materials and Methods

### 2.1. Raw Materials

Nine proso millet cultivars (Cope, Earlybird, Huntsman, Dawn, Rise, Sunrise, Plateau, Minco and Panhandle) were obtained from the University of Nebraska Panhandle Research and Extension Center (Scottsbluff, NE, USA). The cultivars were grown in 2014 at the Dryland Research Farm of the University of Nebraska’s High Plains Agricultural Laboratory (HPAL). Harvest moisture ranged from 7.7% to 10.9%. Seeds from four plots were bulked and samples were randomly collected and stored under appropriate conditions in a grain silo until this study. The experimental work was conducted in 2015; therefore, the grains were analyzed within a short period after harvest. Additional cultivar information, including proximate composition, amylose content, functional properties, pasting properties, and thermal properties, is provided in Supplementary File S2.

Prior to milling, seeds were cleaned using a Ro-Tap sieve shaker (RX-29, W.S. Tyler, Mentor, OH, USA) to remove foreign material. Cleaned grains were dehulled using a modified disk mill (Glenn Mills Inc., Clifton, NJ, USA), in which the stationary disk was replaced with a rubber disk to minimize breakage while ensuring effective hull removal. Dehulled seeds were then milled into flour using a Quadrumat Junior mill (Brabender, Duisburg, Germany).

Whole-wheat flour, corn starch and nonfat dry milk were purchased from Bob’s Red Mill (Milwaukie, OR, USA). Active dry yeast (Fleischmann, St. Louis, MO, USA), shortening (Crisco, Parsippany, NJ, USA), granulated sugar and salt were purchased from a local Walmart store (Lexington, KY, USA). The hydrocolloid used was (courtesy of TIC Gums, White Marsh, MD, USA) a blend of xanthan gum, locust bean gum, carrageenan, and sodium alginate.

### 2.2. Formulation

Gluten-free bread doughs were prepared using a flour base consisting of 50% proso millet flour (cultivar-specific) and 50% corn starch. The remaining ingredients, expressed as baker’s percentages, were TIC 345 hydrocolloid (2%), sugar (8.5%), shortening (4.0%), nonfat dry milk (4.0%), active dry yeast (3.0%), salt (2.0%) and water (105%). All baker’s percentages were calculated on a total dry basis of millet flour + corn starch (100%) [4].

For comparison of bread physical properties (color and texture), a whole-wheat bread control was prepared using the AACC standard method (AACC Method 10-10.03), with a flour to water ratio of 100 g wheat flour to 60 g water [22].

### 2.3. Dynamic Oscillation Measurements

The viscoelastic properties of gluten-free dough were measured using a method described by Singh et al.) using a dynamic oscillatory rheometer (DHR-2, TA Instruments, New Castle, DE, USA) equipped with a 35 mm parallel plate geometry set to a 2 mm gap [4]. All formulation ingredients except yeast were mixed using a 100 g micro mixer (National mfg. Co., Lincoln, NE, USA). The dough was then loaded onto the rheometer plate, the upper plate was lowered to the test gap, and the sample was allowed to rest for 20 min to relax and stabilize prior to testing [4]. To minimize moisture loss during testing, the sample was covered using the rheometer’s solvent trap (evaporation cover) during the equilibration period.

A strain sweep (0.01–100%) was performed at 25 °C and a constant frequency of 0.5 Hz to identify the linear viscoelastic region (LVR). Based on the strain sweep results, a strain amplitude of 0.05% (within the LVR) was selected for subsequent testing [19,23]. Frequency sweeps were then conducted at 25 °C over a range of 0.1–100 Hz to determine the elastic modulus (G′), viscous modulus (G″), and tan δ.

Creep–recovery measurements were conducted using methods described by Dobraszczyk & Morgenstern and Singh et al. [4,19]. Samples were subjected to a constant shear stress of 50 Pa for 60 s (creep phase), followed by stress removal and monitoring for 180 s (recovery phase). Compliance, J(t) (MPa^−1^), and recoverable compliance, J_r_(t) (MPa^−1^), were obtained from the creep–recovery output and used to calculate the following parameters [21,24,25]:

End of creep compliance (J_end_, MPa^−1^):

(1) $$J_{end}=J(t_{c})$$

where t_c_ is the end of the creep phase (~60 s).

Recoverable compliance at the end of recovery (J_r,end_, MPa^−1^):

(2) $$J_{r,end}=J_{r}(t_{r})$$

where t_r_ is the end of the recovery phase (~180 s after unloading).

Elastic fraction (recoverable fraction of deformation):

(3) $$Elastic\;fraction=\frac{J_{r,end}}{J_{end}}$$

Strain recovery (%):

(4) $$\%\;\mathrm{Strain}\;\mathrm{recovery}=\frac{\gamma_{o}-\gamma_{res}}{\gamma_{o}}$$

where γ_o_ is strain immediately after stress removal (first recovery strain value) and γ_res_ is residual strain at end of recovery (last recovery strain value).

Residual Strain (%):

(5) $$\gamma_{res}=\gamma (t_{r})$$

Non-recoverable compliance (J_nr_, MPa^−1^):

(6) $$J_{nr}=J_{end}-J_{r,end}$$

Recovery time to 90% of final recoverable compliance (t_90_, s).

t_90_ was defined as the time during the recovery phase required to reach 90% of the final recovery compliance.

(7) $$J_{r,90}=0.90*J_{r,end}$$

### 2.4. Breadmaking Process

Gluten-free breads were prepared following the procedure described by Singh et al. (2025) [4]. All ingredients were mixed using a kitchen stand mixer (KitchenAid, Model KV25G0X, Benton Harbor, MI, USA) for 1 min at speed 1, followed by 6 min at speed 2. The resulting dough was deposited into baking pans and proofed for 35 min at 40 °C. Loaves were then baked at 190.5 °C for 1 h. After baking, breads were removed from the pans and cooled at room temperature (22 ± 2 °C) for 1 h prior to analysis. A wheat bread control was prepared according to AACC Method 10-10.03 [22]. For storage studies, breads were sealed in Ziploc bags and stored at room temperature (22 ± 2 °C) for 5 days.

### 2.5. Physical Property Determination

#### 2.5.1. Bread Specific Volume

The seed displacement method, as outlined by AACC Method 10-05.01 [22], was used to determine bread volume.

#### 2.5.2. Color

The color of the crust and crumb was determined using a Chromameter (CR-400, Konica Minolta, Ramsey, NJ, USA) using the L* a* b* system. Crust color was measured at six different positions on top of the bread. For crumb color, bread was sliced into three uniform slices of 25 mm, after which color was measured in the center of both slice sides.

### 2.6. Texture Profile Analysis

Bread texture was evaluated according to the Singh et al. (2025) method using a texture analyzer (TA.XTplus, Stable Micro Systems, Surrey, UK) fitted with a 20 mm cylindrical acrylic probe [4]. Bread was sliced into 25 mm-thick slices, and samples were compressed to 40% strain at a crosshead speed of 2.0 mm/s using a trigger force of 20 g. From the resulting force–time curves, firmness (maximum force during the first compression), cohesiveness (ratio of the area under the second compression to the area under the first compression), springiness (ratio of deformation distance recovered between compressions), gumminess (firmness × cohesiveness), and chewiness (firmness × cohesiveness × springiness) were calculated [26].

### 2.7. Data Analysis

All experimental work was conducted in triplicate to provide consistent replication across cultivar formulations. However, it is acknowledged that additional biological and processing replicates would further strengthen the statistical power of cultivar comparisons, particularly for rheological parameters that may be sensitive to sample handling. Results for the study were presented as a mean of three replicates ± standard deviation. Data were analyzed at a significance level of *p* < 0.05 using analysis of variance (ANOVA). For texture profile analysis, the effects of cultivar, storage time, and their interaction were evaluated. When the model effect is significant, mean separation was performed using Tukey’s HSD (Tukey–Kramer when needed) test. Pearson’s correlation analysis was used to evaluate relationships between rheological parameters and bread quality attributes. All statistical analyses were performed using SAS software (Version 9.1.3; SAS Institute Inc., Cary, NC, USA).

## 3. Results and Discussion

### 3.1. Effect of Different Cultivars on Rheological Properties

#### 3.1.1. Strain and Frequency Sweep

The viscoelastic behavior of all formulations was characterized using small-amplitude oscillatory shear (SAOS) and creep–recovery measurements, which are commonly applied to relate gluten-free dough structure to mechanical performance [21]. For oscillatory tests, the linear viscoelastic region (LVR) was determined using a strain amplitude test and 0.05% strain was selected for frequency sweep analysis. Previous studies have reported that wheat flour doughs exhibit linear viscoelasticity at strain levels below 0.1–0.25% [27,28] highlighting the different structural basis of these gluten-free systems, which rely primarily on hydrocolloid- and starch-structured networks rather than gluten networks.

**Figure 1:**
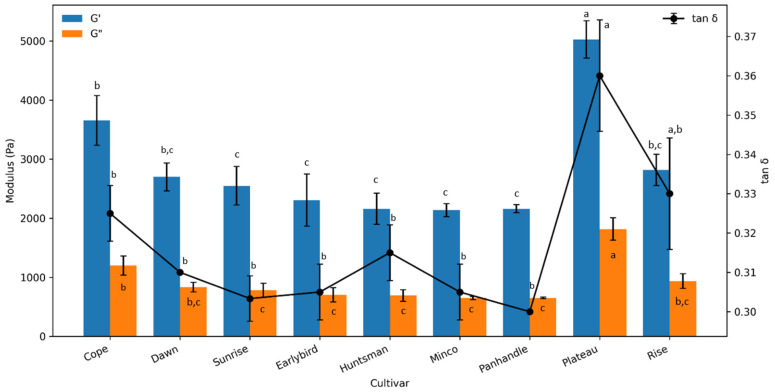
Small-amplitude oscillatory shear parameters (G′, G″ and tan δ) for gluten-free doughs formulated with proso millet cultivars. Values are mean ± standard deviation of three replicates. Means with different letters differ significantly (*p* < 0.05).

The elastic modulus (G′), viscous modulus (G″) and tan(δ) of all formulations within the LVR at 1 Hz frequency are summarized in Figure 1. All formulations exhibited predominantly elastic behavior, as indicated by G’ values exceeding G”, reflecting the formation of weak gel-like structures typical of hydrocolloid-stabilized gluten-free systems. The tan δ values ranged from 0.30 to 0.36, confirming that all doughs behaved as elastic-dominant systems under small deformation.

The Plateau millet cultivar showed the highest G′ and G″, indicating a firm dough structure, while cultivars such as Minco, Panhandle and Huntsman showed comparatively lower moduli. Although all samples remained elastic-dominant (tan δ < 1), Plateau also showed a higher tan δ, suggesting greater viscous behavior (higher energy dissipation) relative to the other cultivars. The observed differences are consistent with starch’s compositional effects, particularly amylose–amylopectin functionality. Cultivars with lower amylose, particularly waxy types, exhibited enhanced swelling and water-binding capacity due to the predominance of amylopectin, which contributed to increased viscoelastic moduli. Plateau is a waxy cultivar, while Cope represents the lowest-amylose non-waxy cultivar among the nine cultivars [16]. Consistent with this mechanism in the same study by Singh et al. [16], Plateau showed the highest swelling power and solubility, and both Plateau and Cope also exhibited the highest water binding capacity among all the cultivars, supporting stronger hydration-driven structuring. In line with this, strong negative correlations were observed between amylose content and G′ (r = −0.91) and amylose content and G″ (r = −0.92), indicating that decreasing amylose is associated with a firmer, more structured dough.

In gluten-free doughs, the viscoelastic network is not formed by gluten but instead develops through interactions among starch granules, hydrocolloids, proteins and water [4,19,29]. The hydrocolloid blend used in this formulation likely contributed to water immobilization and continuous phase viscosity, while cultivar-specific millet starch properties influenced swelling, hydration and resistance to deformation [16,17,29]. Therefore, the observed differences in G′ and G″ reflect not only total dough stiffness but also the degree to which each cultivar interacted with the hydrocolloid–starch matrix.

From a functional perspective, a higher G′ indicates greater resistance to deformation and may support gas cell stability during proofing. However, excessive stiffness can restrict gas cell expansion and reduce oven spring [30,31]. Therefore, the low specific volume observed for Plateau may reflect excessive stiffness and early structure setting, whereas the higher volumes of Rise and Earlybird suggest a more favorable balance between gas retention and dough expansion.

During baking, starch gelatinization and hydrocolloid-mediated water distribution contribute to crumb setting. Cultivar-dependent starch swelling and amylose–amylopectin behavior may therefore affect the timing and strength of structure formation. These mechanisms help explain why differences in small deformation rheology were associated with loaf expansion and later texture stability. This interpretation is consistent with Dobraszczyk, who emphasized that gas cell rheological stability is critical for gas retention and final loaf volume [30]. Similarly, Ren et al. (2020) reported in gluten-free breads that overly firm and low-extensibility dough structures can prohibit gas cell growth and reduce loaf volume [31].

#### 3.1.2. Creep and Recovery

**Figure 2:**
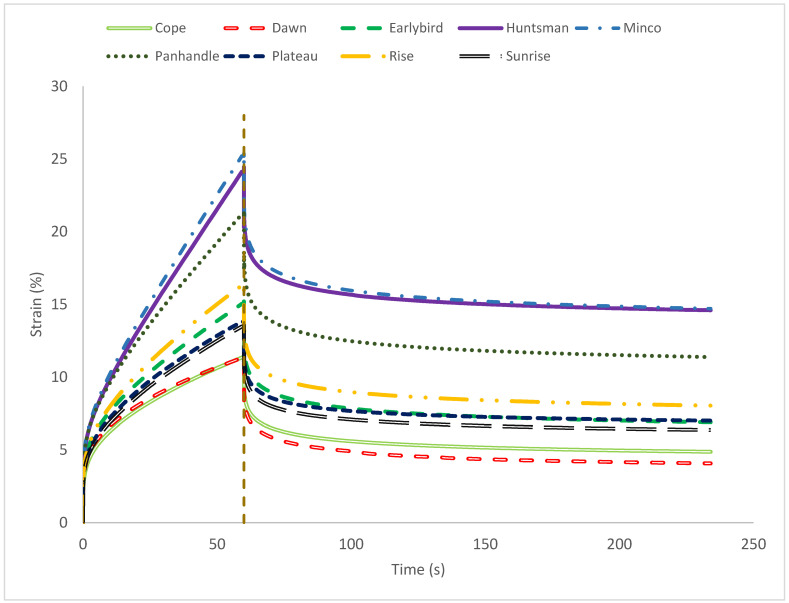
Creep–recovery compliance (J, MPa^−1^) profiles for gluten-free doughs prepared from nine proso millet cultivars. The vertical dashed line marks the end of the creep phase and the start of recovery (*n* = 3).

Creep–recovery curves (Figure 2) confirmed that all gluten-free doughs exhibited viscoelastic behavior, showing an immediate strain increase upon loading (instantaneous compliance), continued strain growth during the creep phase (time-dependent flow), and partial strain recovery after stress removal. In general, higher creep strain (or higher compliance) indicates lower resistance to deformation, while the extent of recovery reflects the ability of the dough structure to elastically rebound rather than permanently flow [32].

Across cultivars, the compliance at the end of the creep phase (J_end_) varied substantially (Table 1), indicating cultivar-dependent resistance to deformation. Minco exhibited the highest J_end_ (5041 MPa^−1^), followed by Huntsman (4055 MPa^−1^) and Panhandle (3936 MPa^−1^), consistent with the higher peak strains observed in Figure 2. These cultivars therefore showed the lowest resistance to deformation under constant stress. In contrast, Dawn (2394 MPa^−1^) and Cope (2463 MPa^−1^) showed the lowest J_end_ values, indicating greater resistance to creep deformation. The deformation was divided into recoverable and non-recoverable compliance, further differentiating cultivars. The non-recoverable compliance represents the viscous/irreversible part of deformation. Minco had the highest J_nr_ (2874 MPa^−1^) and the highest residual strain (14.71%), indicating a greater tendency for permanent flow and structural rearrangement after stress removal. Conversely, Dawn showed the lowest J_nr_ (898 MPa^−1^) and lowest residual strain (4.58%), suggesting greater resistance to irreversible deformation. Although elastic fraction (J_r_/J) and strain recovery (%) were not statistically different among cultivars, the numerical trend followed a similar trend where Dawn had the highest elastic fraction (0.626) and recovery (62%), while Minco had the lowest elastic fraction (0.431) and recovery (43%). Recovery kinetics were broadly similar among cultivars, as t_90_ ranged from 38 to 48 s but did not differ significantly (Table 1), implying that once stress was removed, the rate at which recovery approached completion was comparable among cultivars, even though the amount of permanent deformation differed.

From a functional standpoint, cultivars with lower J_end_ and J_nr_ and lower residual strain (Dawn) are expected to better maintain structure under sustained stresses during handling and proofing, whereas cultivars with higher J_nr_ and residual strain (Minco) may be more prone to shape loss and irreversible deformation. Since higher recovery has been associated with improved loaf volume in some gluten-free systems [33], the recovery-related metrics (elastic fraction/recovery) suggest potential advantages for cultivars such as Dawn; however, optimal baking performance likely depends on achieving a balance between structural stability (low irreversible deformation) and sufficient deformability/extensibility to allow expansion during oven spring. Similar creep–recovery approaches have been used to evaluate hydrocolloid-structured gluten-free doughs, where hydrocolloid type and level can shift compliance and recovery behavior [4,34], highlighting that cultivar starch functionality and formulation matrix can alter how hydrocolloid-structured systems deform and recover.

### 3.2. Effect of Cultivar on Millet Bread Physical Properties

#### 3.2.1. Bread Color

Cultivar formulation significantly influenced crust lightness (L*) and redness (a*) (*p* < 0.05) (Table 2; representative loaf photographs are provided in the Supplementary Materials). Across gluten-free breads, crust L* ranged from 61.89 to 68.35, with Plateau producing the lightest crust (L* = 68.35) and cultivars such as Huntsman, Minco, and Rise showing comparatively lower L* values. Crust a* ranged from −0.35 to 1.76, with Plateau showing the lowest redness (a* = −0.35) and Dawn among the highest (a* = 1.76), indicating cultivar-dependent differences in surface color development. In contrast, crust b* values for gluten-free breads were relatively similar (~30.7–33.0) and did not differ significantly among cultivars, suggesting comparable yellowness once baked. Despite these cultivar effects, all gluten-free loaves exhibited substantially lighter and less red crusts than wheat bread (wheat: L* = 43.86, a* = 9.41), resulting in large crust color differences relative to wheat. Similar lighter crusts and lower crust redness in gluten-free breads compared with wheat controls have been reported in other gluten-free systems, supporting that formulation matrix and available browning precursors can shift crust color development away from wheat-like browning [35]. These differences likely reflect the fundamentally different formulation matrix and browning chemistry of gluten-free systems compared with wheat, while the cultivar-related shifts in L* and a* may be associated with differences in flour or starch composition, surface moisture loss, and crust-setting dynamics during baking [36].

Crumb color showed clearer cultivar-driven differences. Crumb L* ranged from 63.11 to 70.17, with Minco producing the lightest crumb (L* = 70.17) and Plateau producing the darkest crumb (L* = 63.11). The dark crumb coloration of Plateau cultivar may be associated with the waxy starch-linked structural effects, consistent with whole waxy wheat bread showing reduced volume and lower crumb lightness (L*) with increasing waxy flour inclusion [37]. Crumb a* values were consistently negative (more green/neutral than crust), and crumb b* ranged from 18.85 to 23.48, with Plateau and Minco among the highest b* values, indicating a more yellow crumb tone. Crumb ΔE values (relative to wheat) ranged from 11.40 to 16.86, confirming that crumb appearance also differed substantially from wheat bread. Interestingly, the waxy cultivar Plateau produced the lightest crust (highest L*) but the darkest crumb (lowest L*). This contrast likely reflects different drivers of color development, crust color is largely controlled by surface browning and dehydration, whereas crumb lightness is strongly influenced by internal porosity and light scattering within the cellular crumb matrix [36]. In line with this interpretation, crumb L* showed a positive trend with specific volume (r = 0.62), and Plateau’s low loaf volume (denser crumb) may have reduced light scattering and lowered crumb L* despite a lighter crust [38].

#### 3.2.2. Bread Volume

The specific volume of gluten-free breads differed significantly among cultivar formulations and was consistently lower than the whole-wheat control (*p* < 0.05) (Table 2). Whole-wheat bread exhibited a specific volume of 3.58 mL/g, whereas proso millet breads ranged from 1.97 to 2.43 mL/g, reflecting the absence of a gluten network and the reliance on starch–hydrocolloid structuring for gas retention during proofing and baking. Among cultivars, Rise (2.43 mL/g) and Earlybird (2.40 mL/g) produced the highest volumes and were comparable to Panhandle (2.37 mL/g), Minco (2.32 mL/g), and Huntsman (2.29 mL/g). In contrast, Plateau produced the lowest specific volume (1.97 mL/g), indicating poorer expansion and reduced gas-holding capacity.

Across cultivars, specific volume showed a significant negative correlation with viscoelastic moduli, including G′ (r = −0.80, *p* = 0.009) and G″ (r = −0.79, *p* = 0.011), suggesting that excessively rigid dough structures may limit gas cell expansion during baking [4]. The full correlation matrix is provided in the Supplementary Materials. In addition, low-amylose (waxy) behavior was associated with reduced loaf volume, and a strong positive relationship was observed between amylose content and specific volume (r = 0.82), indicating that cultivar starch composition contributed to differences in expansion and structure holding during baking. Higher amylopectin systems can absorb water rapidly and swell extensively during heating, which may weaken cell-wall integrity and reduce the dough’s ability to maintain gas cells as they expand, leading to lower loaf volumes [3,16]. Creep–recovery metrics describing irreversible deformation (J_end_, J_nr_, residual strain) were not significantly correlated with specific volume (*p* > 0.05). However, recovery time scale (t_90_) covaried positively with specific volume (r = 0.88, *p* = 0.0018). Although t_90_ did not differ significantly among cultivars (Table 1), its positive correlation with specific volume suggests that recovery time scale may be related to loaf expansion. Hydrocolloids can increase dough viscosity and stabilize gas cells [29]; however, excessive viscosity and early setting can restrict expansion, as reported for gluten-free systems with increasing HPMC [39] and xanthan gum [14]. Overall, the cultivar-dependent specific volume results likely reflect a balance between viscosity-driven gas retention and sufficient deformability/extensibility to allow oven spring.

### 3.3. Impact of Cultivar on Textural Properties

**Figure 3:**
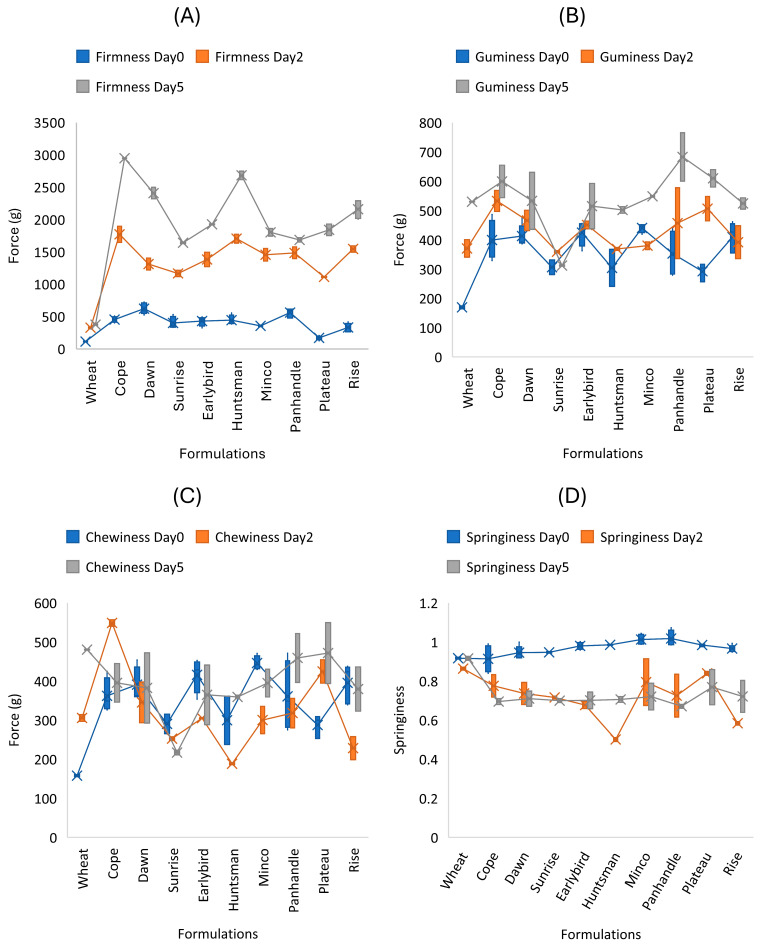

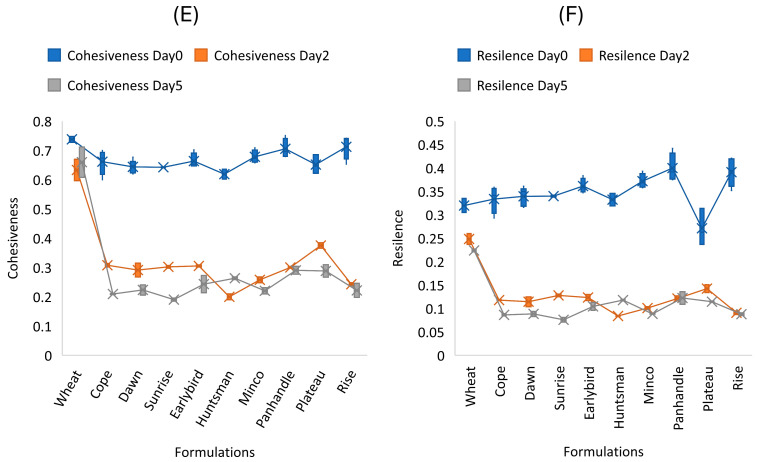
Effect of storage time (0, 2 and 5 days) on texture analysis (TPA) parameters of gluten-free bread formulated with proso millet cultivars: (**A**) firmness, (**B**) gumminess, (**C**) chewiness, (**D**) springiness, (**E**) cohesiveness, (**F**) resilience. Error bars represent standard deviation.

Texture profile analysis (TPA) showed that both cultivar and storage time (0, 2, and 5 days) strongly influenced the crumb texture of the gluten-free breads (Figure 3). Overall, storage induced a typical staling response, characterized by increased crumb firmness and reduced elastic recovery, likely associated with starch retrogradation and moisture redistribution. Firmness (Figure 3A) increased significantly from Day 0 to Day 5 for all proso millet breads, demonstrating rapid firming during storage. Differences among cultivars were apparent, with Cope and Huntsman showing the highest firmness by Day 5, while Sunrise remained comparatively softer. This firming behavior is consistent with starch retrogradation and moisture redistribution during storage, which increases crumb rigidity and the force required to compress the crumb [17]. Although firmness changes during storage are commonly associated with starch retrogradation and moisture redistribution, retrogradation was not directly measured using DSC or XRD in the present study. Therefore, this interpretation should be considered mechanistic but indirect. Gumminess (Figure 3B) increased with storage, indicating that more energy was required to disintegrate the crumb as it aged. By Day 5, Panhandle, Plateau and Cope showed relatively high gumminess values, whereas Sunrise remained comparatively lower, aligning with its lower firmness trend. Chewiness (Figure 3C) displayed cultivar-dependent changes with storage rather than a uniform increase, reflecting the competing effects of rising firmness and declining elastic recovery. Several cultivars showed higher chewiness at Day 5 compared with Day 0, while others exhibited smaller changes or intermediate behavior.

Elastic recovery parameters consistently declined during storage. Springiness (Figure 3D) decreased after Day 0, indicating reduced rebound after compression. Cohesiveness (Figure 3E) dropped sharply from Day 0 to Day 2 and remained low through Day 5 for all gluten-free breads, suggesting increased crumb fragility and a greater tendency to fracture during compression. Similarly, resilience (Figure 3F) decreased strongly with storage, confirming reduced instantaneous elastic recovery and a progressively more rigid crumb structure.

Among cultivars, amylose content correlated positively with gumminess (r = 0.67), chewiness (r = 0.73), and resilience (r = 0.83), indicating that higher-amylose cultivars tended to produce crumbs that were more resistant to breakdown and exhibited greater elastic recovery. These trends are consistent with prior gluten-free studies showing that increase in viscosity can elevate crumb firmness, such as the reported increase in crumb hardness with increasing xanthan gum concentration in sorghum-based gluten-free breads [14] and emphasize that cultivar-driven starch composition can shift the balance between initial texture and storage stability.

### 3.4. Relationships Between Texture and Viscoelastic Behavior

To relate crumb texture to viscoelastic behavior, Pearson’s correlations were evaluated between TPA parameters (Supplementary File S1) and (i) small-deformation oscillatory rheology (G′, G″, tan δ), (ii) creep–recovery parameters. A strong linkage was observed between bread volume and elastic recovery, Day 0 resilience was positively correlated with specific volume (r = 0.92), indicating that formulations yielding greater expansion also produced crumbs with higher instantaneous elastic recovery, consistent with a more open crumb structure. This agrees with prior studies that loaf volume strongly influences crumb mechanical properties and elasticity, consistent with a cellular-solid description of bread crumb in which a continuous cell-wall matrix surrounding gas cells governs compression response [40,41]. Similarly, Day 2 chewiness was positively correlated to dough elasticity (G′) (r = 0.66), supporting a structure-setting interpretation in which a more elastic, structured dough corresponds to a crumb that is mechanically more resistant to breakdown early in storage. In gluten-free bread systems, Demirkesen et al. [13] reported significant correlations between dough viscoelastic moduli and rice-bread firmness, linking dough rheology directly to baked crumb mechanical response. Researchers also found that gluten-free doughs with higher viscoelastic moduli and lower creep compliance produced breads with lower specific volumes and an overall harder crumb [42].

By Day 5, absolute TPA values were more strongly governed by staling processes common to starch-based crumbs; therefore, retention metrics were more informative. In particular, Δspringiness (Day 5 − Day 0) covaried with dough elastic modulus, G′ (r = 0.72), indicating that more structured viscoelastic systems tended to retain springiness more effectively during storage. Consistent with prior gluten-free work showing that increased dough rigidity (higher G′/G″ and lower creep compliance) corresponds to increased mechanical strength of the baked product, cultivar-dependent differences in dough structure here were reflected in crumb texture and its retention during storage [21].

Creep–recovery metrics showed clearer relationships with fresh crumb mechanics (Day 0) and with storage-related changes. At Day 0, the recovery time scale (t_90_) was positively correlated with chewiness (r = 0.88) and gumminess (r = 0.81), and it also covaried with resilience (r = 0.79). In addition, Day 0 springiness correlated with compliance-based creep measures (J_end_: r = 0.79; J_nr_: r = 0.73; residual strain: r = 0.73), suggesting that cultivar-dependent and time-dependent deformation behavior in the dough phase is reflected in baked crumb rebound characteristics. Prior studies showed that recovery behavior and kinetics from creep–recovery are strongly associated with crumb texture attributes reinforcing the idea that recovery-dominated metrics capture structural elasticity relevant to the baked product [33,43]. During storage, creep parameters were more informative when expressed as changes over time, higher J_end_ was associated with Δspringiness (Day 5 − Day 0) (r = −0.69), and higher t_90_ was associated with a decline in resilience during storage (Δresilience; r = −0.69). Collectively, these relationships indicate that both small-deformation rheology and creep–recovery behavior capture cultivar-dependent structural attributes that manifest in fresh crumb texture and in the retention of elastic properties during staling. Prior work comparing shear creep–recovery with compression–recovery shows that creep parameters often align with softening/deformation behavior, whereas recovery-based large-deformation metrics can be more sensitive to structural differences [25].

## 4. Conclusions

Proso millet cultivar significantly influenced gluten-free bread rheology, loaf expansion, and textural stability during storage. Low-amylose (waxy) starch functionality, exemplified by Plateau, produced stiffer doughs (higher G′ and G″), lower specific volumes, and a denser crumb with reduced lightness, whereas higher-amylose cultivars generally yielded less firm doughs, greater loaf expansion, and improved elastic recovery. Correlation analysis indicated that oscillatory rheology was most strongly associated with early-storage mechanical behavior (Day 2 chewiness), while creep–recovery descriptors, particularly t_90_ and compliance metrics, aligned with fresh-crumb rebound and the retention of elastic attributes (springiness and resilience) during staling. Collectively, these results show that cultivar-dependent starch structure governs both dough viscoelasticity and staling-related texture evolution, highlighting cultivar selection as a practical route to improving gluten-free bread quality and shelf-life consistency. Future studies incorporating DSC or XRD could further expand the mechanistic understanding of cultivar-dependent texture changes during storage.

## Figures and Tables

**Table 1:** Creep and recovery parameters of gluten-free dough formulated with proso millet cultivars.

Cultivar	J_end_ (MPa^−1^)	Elastic Fraction (J_r_/J)	Strain Recovery (%)	Residual Strain (%)	J_nr_ (MPa^−1^)	t_90_ (s)
Cope	2463.2 ± 78.0 ^b^	0.520 ± 0.013 ^a^	51.52 ± 1.33 ^a^	6.02 ± 0.03 ^ab^	1182.5 ± 4.5 ^ab^	43.08 ± 3.52 ^a^
Dawn	2393.5 ± 141.8 ^b^	0.626 ± 0.041 ^a^	62.13 ± 4.04 ^a^	4.58 ± 0.76 ^b^	898.0 ± 150.2 ^b^	45.05 ± 0.74 ^a^
Sunrise	2696.9 ± 282.4 ^b^	0.539 ± 0.017 ^a^	53.31 ± 1.75 ^a^	6.37 ± 0.91 ^ab^	1246.9 ± 176.5 ^ab^	39.28 ± 0.37 ^a^
Earlybird	3007.0 ± 580.9 ^b^	0.565 ± 0.127 ^a^	55.91 ± 12.67 ^a^	6.89 ± 3.24 ^ab^	1344.3 ± 634.4 ^ab^	47.15 ± 5.19 ^a^
Huntsman	4054.9 ± 4.5 ^ab^	0.469 ± 0.023 ^a^	46.34 ± 2.24 ^a^	11.01 ± 0.44 ^ab^	2153.4 ± 90.1 ^ab^	44.00 ± 0.74 ^a^
Minco	5041.1 ± 267.2 ^a^	0.431 ± 0.040 ^a^	42.52 ± 3.89 ^a^	14.71 ± 1.76 ^a^	2873.7 ± 352.6 ^a^	47.67 ± 1.48 ^a^
Panhandle	3936.0 ± 938.8 ^ab^	0.528 ± 0.118 ^a^	52.23 ± 11.77 ^a^	9.79 ± 4.63 ^ab^	1912.9 ± 908.0 ^ab^	45.70 ± 10.20 ^a^
Plateau	2761.3 ± 227.9 ^b^	0.505 ± 0.046 ^a^	49.97 ± 4.41 ^a^	7.01 ± 1.17 ^ab^	1371.6 ± 240.9 ^ab^	37.97 ± 2.97 ^a^
Rise	3570.7 ± 780.5 ^ab^	0.500 ± 0.080 ^a^	49.41 ± 8.00 ^a^	9.30 ± 3.46 ^ab^	1817.8 ± 675.2 ^ab^	46.10 ± 3.71 ^a^

Note: Values are mean ± standard deviation of three replicates. Means with different letters in a column differ significantly (*p* < 0.05). J_end_ is the compliance at the end of the creep phase; J_r_ is the recoverable (elastic) compliance; J_nr_ is the non-recoverable (viscous) compliance (J_nr_ = J_end_ − J_r_). t_90_ is the time required to reach 90% of recovery during the recovery phase.

**Table 2:** Effect of cultivar formulations on specific volume of bread and crust and crumb color of gluten-free bread.

Formula	Specific Volume	Crust Color				Crumb Color			
	mL/g	L*	a*	b*	ΔE	L*	a*	b*	ΔE
Wheat	3.58 ± 0.03 ^a^	43.86 ± 2.02 ^c^	9.41 ± 0.39 ^a^	25.35 ± 1.61 ^b^	0.00	57.81 ± 4.10 ^e^	2.52 ± 0.46 ^a^	26.59 ± 1.34 ^a^	0.00
Cope	2.19 ± 0.05 ^d,e^	65.89 ± 3.73 ^a,b^	0.36 ± 1.79 ^b,c^	31.41 ± 4.56 ^a^	24.58	69.93 ± 1.91 ^a,b^	−6.28 ± 0.24 ^b^	18.85 ± 0.58 ^f^	16.86
Dawn	2.22 ± 0.05 ^c,d,e^	63.82 ± 4.94 ^a,b^	1.76 ± 1.21 ^b^	33.02 ± 2.94 ^a^	22.71	68.54 ± 1.66 ^a-c^	−6.32 ± 0.18 ^b^	20.82 ± 0.64 ^d,e^	15.05
Sunrise	2.17 ± 0.02 ^e^	66.15 ± 6.06 ^a,b^	1.50 ± 2.39 ^b^	32.78 ± 3.16 ^a^	24.79	67.49 ± 2.24 ^b,c^	−7.00 ± 0.29 ^c,d^	21.85 ± 1.11 ^c^	14.38
Earlybird	2.40 ± 0.05 ^b^	65.04 ± 4.78 ^a,b^	0.36 ± 0.94 ^b,c^	31.38 ± 3.00 ^a^	23.81	67.70 ± 2.58 ^a-c^	−6.78 ± 0.23 ^c^	21.37 ± 0.75 ^c,d^	14.54
Huntsman	2.29 ± 0.08 ^b-e^	61.89 ± 6.93 ^b^	1.47 ± 2.48 ^b^	31.84 ± 4.63 ^a^	20.74	66.16 ± 2.58 ^c^	−7.09 ± 0.25 ^d^	20.94 ± 0.99 ^d,e^	13.93
Minco	2.32 ± 0.01 ^b,c,d^	62.27 ± 7.90 ^b^	0.95 ± 0.70 ^b,c^	31.51 ± 4.19 ^a^	21.18	70.17 ± 1.25 ^a^	−7.67 ± 0.15 ^e^	23.23 ± 0.80 ^b^	16.37
Panhandle	2.37 ± 0.08 ^b,c^	65.31 ± 4.68 ^a,b^	0.53 ± 1.42 ^b,c^	31.42 ± 3.01 ^a^	24.00	68.97 ± 2.77 ^a,b^	−6.90 ± 0.28 ^c,d^	19.33 ± 0.79 ^f^	16.31
Plateau	1.97 ± 0.01 ^f^	68.35 ± 5.29 ^a^	−0.35 ± 0.81 ^c^	30.72 ± 2.96 ^a^	26.90	63.11 ± 2.91 ^d^	−7.08 ± 0.33 ^d^	23.48 ± 1.08 ^b^	11.40
Rise	2.43 ± 0.17 ^b^	62.78 ± 6.67 ^b^	1.17 ± 1.31 ^b^	31.20 ± 3.50 ^a^	21.45	68.54 ± 2.38 ^a-c^	−6.50 ± 0.22 ^b^	20.42 ± 0.60 ^e^	15.32

Note: Values are mean ± standard deviation of three replicates. Means with different letters in a column differ significantly (*p* < 0.05). ΔE was calculated relative to the wheat control using ΔE = sqrt[(ΔL*)^2^ + (Δa*)^2^ + (Δb*)^2^].

## Data Availability

The original contributions presented in this study are included in the article/Supplementary Materials. Further inquiries can be directed to the corresponding author.

## Supplementary Materials

The following supporting information can be downloaded at: https://www.mdpi.com/article/10.3390/foods15101711/s1, File S1: Correlation data in Excel format. File S2: Tables S1–S4 (word format), including Table S1: Approximate content of proso millet cultivars; Table S2: Functional properties of cultivars; Table S3: Pasting properties of proso millet cultivars; Table S4: Thermal gelatinization properties of proso millet cultivars. File S3: Figure S1 (word format): Photographs of gluten-free breads prepared from nine proso millet cultivars (cross-section, side-view, and top-view).
