# Supplementary material for: Proso Millet Cultivar Effects on Rheology of Dough and Quality Characteristics of Gluten-Free Breads
Source: Foods. 2026 May 13;15(10):1711. doi: 10.3390/foods15101711 (PMC13206684; doi:10.3390/foods15101711)
Supplement: Supplementary file 1 [file foods-15-01711-s001.zip › File S2_Cultivar composition, amylose content, functional properties, pasting properties, and thermal properties.pdf]

Proso Millet Cultivar Effects on Rheology of Dough and Quality Characteristics of Gluten-Free Breads

Manjot Singh and Akinbode A. Adedeji *

Table S1: Proximate content of proso millet cultivars

Cultivar	Moisture (%)	Crude Protein (%)	Crude Fat (%)	Crude Fiber (%)	Ash (%)	Carbohydrate (%)	Amylose (%)
Cope	10.29 ± 0.02^{b}	14.38 ± 0.06^{c}	2.01 ± 0.08^{b}	0.57 ± 0.06^{a}	0.68 ± 0.02^{d}	82.86 ± 0.12^{a}	18.15 ± 1.06^{f}
Dawn	9.40 ± 0.08^{d}	15.14 ± 0.01^{a}	3.51 ± 0.03^{a}	0.59 ± 0.30^{a}	0.77 ± 0.01^{bc}	80.50 ± 0.06^{c}	25.10 ± 0.28^{c}
Earlybird	10.18 ± 0.06^{b}	13.78 ± 0.05^{d}	3.60 ± 0.13^{a}	0.94 ± 0.35^{a}	0.83 ± 0.08^{bc}	81.70 ± 0.10^{b}	30.20 ± 0.57^{b}
Huntsman	10.18 ± 0.15^{b}	13.48 ± 0.08^{e}	3.67 ± 0.11^{a}	0.90 ± 0.04^{a}	0.84 ± 0.02^{bc}	81.92 ± 0.20^{ab}	21.40 ± 0.57^{e}
Minco	9.64 ± 0.11^{cd}	13.39 ± 0.01^{e}	3.45 ± 0.23^{a}	0.71 ± 0.06^{a}	0.82 ± 0.00^{bc}	82.24 ± 0.25^{a}	34.60 ± 0.28^{a}
Panhandle	10.35 ± 0.03^{b}	14.34 ± 0.03^{c}	3.85 ± 0.04^{a}	0.84 ± 0.28^{a}	0.90 ± 0.04^{ab}	80.88 ± 0.02^{bc}	26.40 ± 0.57^{c}
Plateau	9.71 ± 0.13^{c}	14.79 ± 0.02^{b}	3.63 ± 0.13^{a}	0.80 ± 0.08^{a}	0.74 ± 0.00^{cd}	80.66 ± 0.11^{bc}	3.10 ± 0.28^{g}
Rise	10.71 ± 0.01^{a}	12.43 ± 0.01^{f}	3.26 ± 0.76^{a}	0.93 ± 0.11^{a}	0.99 ± 0.02^{a}	83.20 ± 0.79^{a}	25.75 ± 0.07^{cd}
Sunrise	9.45 ± 0.09^{cd}	14.20 ± 0.01^{c}	3.59 ± 0.07^{a}	0.81 ± 0.01^{a}	0.98 ± 0.01^{a}	81.12 ± 0.05^{b}	24.40 ± 1.41^{d}

Note: Values are means ± standard deviations of two replicates. Crude protein, crude fat, ash, and carbohydrate are reported on a dry basis. Carbohydrate was calculated as: 100 − (% protein + % fat + % ash). Means in the same column with different letters are significantly different at $p < 0.05$. Data adapted from: Singh, M.; Adedeji, A.; Santra, D. Physico-chemical and functional properties of nine proso millet cultivars. Transactions of the ASABE 2018, 61(3), 1165–1174. https://doi.org/10.13031/trans.12553

Table S2: Functional properties of cultivars.

Cultivar	Water Binding Capacity (%)	70°C		80°C		90°C	
		Solubility (%)	Swelling (%)	Solubility (%)	Swelling (%)	Solubility (%)	Swelling (%)
Cope	236.92 ± 0.70[a]	6.95 ± 0.49[b]	8.12 ± 0.48[ab]	21.15 ± 0.64[b]	11.13 ± 0.16[d]	50.45 ± 1.48[b]	22.32 ± 3.80[cd]
Dawn	206.50 ± 0.35[c]	3.85 ± 0.07[c]	7.36 ± 0.14[b]	8.25 ± 0.07[cd]	14.14 ± 0.77[bc]	46.75 ± 0.78[bc]	30.36 ± 3.20[ab]
Earlybird	207.79 ± 1.38[c]	3.30 ± 0.14[c]	6.75 ± 0.04[b]	7.10 ± 1.41[de]	12.76 ± 2.45[bcd]	19.20 ± 4.10[ef]	21.65 ± 2.03[cd]
Huntsman	206.99 ± 0.60[c]	3.75 ± 0.07[c]	8.11 ± 0.08[ab]	7.05 ± 0.21[de]	13.17 ± 0.86[bcd]	23.50 ± 2.97[de]	26.00 ± 1.77[bcd]
Minco	224.87 ± 1.10[b]	4.05 ± 0.21[c]	8.35 ± 0.29[ab]	6.40 ± 0.28[e]	11.76 ± 0.13[cd]	42.30 ± 4.81[c]	27.56 ± 1.64[abc]
Panhandle	207.56 ± 3.18[c]	3.85 ± 0.07[c]	7.41 ± 0.21[b]	8.30 ± 0.01[cd]	14.19 ± 0.70[bc]	46.80 ± 0.84[bc]	30.41 ± 3.12[ab]
Plateau	235.49 ± 1.85[a]	15.00 ± 2.97[a]	9.86 ± 3.01[a]	61.65 ± 0.07[a]	23.73 ± 0.92[a]	70.35 ± 4.31[a]	24.67 ± 2.29[bcd]
Rise	201.95 ± 1.46[d]	4.60 ± 0.14[c]	7.86 ± 0.09[ab]	9.40 ± 0.57[c]	15.16 ± 1.22[b]	30.50 ± 4.24[d]	34.37 ± 6.16[a]
Sunrise	208.97 ± 0.26[c]	3.75 ± 0.07[c]	7.26 ± 0.51[b]	6.75 ± 0.64[e]	11.18 ± 0.68[d]	13.80 ± 0.71[f]	19.51 ± 2.96[d]

Note: Values are means ± standard deviations of three replicates. Means in the same column with different letters are significantly different at $p < 0.05$. Data adapted from: Singh, M.; Adedeji, A.; Santra, D. Physico-chemical and functional properties of nine proso millet cultivars. *Transactions of the ASABE* **2018**, *61*(3), 1165–1174. https://doi.org/10.13031/trans.12553

Table S3. Pasting properties of proso millet cultivars

Cultivar	Pasting Temperature (°C)	Peak Viscosity (Pa·s)	Holding Strength (Pa·s)	Final Viscosity (Pa·s)	Breakdown (Pa·s)	Setback (Pa·s)
Cope	77.52 ± 0.10[f]	1.05 ± 0.02[e]	0.52 ± 0.01[d]	1.49 ± 0.03[d]	0.53 ± 0.02[d]	0.97 ± 0.03[e]
Dawn	82.05 ± 0.81[c]	1.62 ± 0.01[c]	0.74 ± 0.01[c]	2.84 ± 0.04[b]	0.88 ± 0.01[b]	2.10 ± 0.04[b]
Earlybird	79.06 ± 0.39[e]	1.91 ± 0.03[a]	0.83 ± 0.01[b]	3.15 ± 0.01[a]	1.08 ± 0.04[a]	2.32 ± 0.01[a]
Huntsman	77.31 ± 0.77[f]	1.80 ± 0.01[b]	0.77 ± 0.01[c]	2.24 ± 0.01[c]	1.03 ± 0.03[a]	1.47 ± 0.02[d]
Minco	80.56 ± 0.75[d]	1.89 ± 0.04[ab]	0.84 ± 0.03[b]	2.88 ± 0.04[b]	1.05 ± 0.07[a]	2.04 ± 0.01[b]
Panhandle	80.49 ± 0.51[d]	1.53 ± 0.01[d]	0.87 ± 0.01[ab]	2.81 ± 0.01[b]	0.66 ± 0.01[c]	1.94 ± 0.01[c]
Plateau	76.76 ± 0.03[f]	0.92 ± 0.01[f]	0.43 ± 0.01[e]	0.71 ± 0.01[e]	0.49 ± 0.01[d]	0.28 ± 0.01[f]
Rise	88.87 ± 0.23[a]	1.93 ± 0.05[a]	0.90 ± 0.02[a]	3.22 ± 0.01[a]	1.03 ± 0.04[a]	2.32 ± 0.02[a]
Sunrise	87.31 ± 0.16[b]	1.62 ± 0.08[c]	0.73 ± 0.04[c]	2.80 ± 0.13[b]	0.89 ± 0.05[b]	2.06 ± 0.09[b]

Note: Values are means ± standard deviations of three replicates. Means in the same column with different letters are significantly different at $p < 0.05$. Data adapted from: Singh, M.; Adedeji, A.; Santra, D. Physico-chemical and functional properties of nine proso millet cultivars. *Transactions of the ASABE* **2018**, *61*(3), 1165–1174. https://doi.org/10.13031/trans.12553

Table S4. Thermal gelatinization properties of proso millet cultivars

Cultivar	Onset Temperature (°C)	Peak Temperature (°C)	End Temperature (°C)	ΔH_G (J/g)	Range (°C)
Cope	71.85 ± 0.01^{c}	78.32 ± 0.17^{b}	91.80 ± 0.49^{ab}	2.65 ± 0.39^{bcd}	19.95 ± 0.51^{a}
Dawn	71.62 ± 0.02^{cd}	77.22 ± 0.15^{c}	91.78 ± 1.54^{ab}	2.51 ± 0.22^{bcd}	19.17 ± 2.93^{a}
Earlybird	71.32 ± 0.01^{d}	76.49 ± 0.12^{d}	88.95 ± 0.60^{c}	2.41 ± 0.08^{cd}	17.63 ± 0.59^{a}
Huntsman	72.57 ± 0.50^{b}	77.84 ± 0.64^{bc}	91.92 ± 0.17^{ab}	2.43 ± 0.16^{cd}	19.36 ± 0.33^{a}
Minco	70.59 ± 0.01^{e}	75.66 ± 0.15^{e}	89.38 ± 1.84^{c}	2.91 ± 0.36^{b}	17.79 ± 3.26^{a}
Panhandle	71.90 ± 0.28^{c}	77.20 ± 0.14^{c}	92.13 ± 0.58^{ab}	2.88 ± 0.03^{bc}	20.23 ± 0.30^{ab}
Plateau	74.27 ± 0.09^{a}	79.41 ± 0.01^{a}	92.53 ± 1.58^{ab}	3.45 ± 0.09^{a}	18.26 ± 1.67^{a}
Rise	71.59 ± 0.01^{cd}	76.54 ± 0.15^{d}	90.19 ± 0.18^{bc}	2.52 ± 0.08^{bcd}	18.60 ± 0.19^{a}
Sunrise	72.38 ± 0.21^{b}	78.14 ± 0.42^{b}	92.64 ± 0.67^{a}	2.38 ± 0.18^{d}	20.26 ± 0.88^{a}

Note: Values are means ± standard deviations of three replicates. Means in the same column with different letters are significantly different at $p < 0.05$. Data adapted from: Singh, M.; Adedeji, A.; Santra, D. Physico-chemical and functional properties of nine proso millet cultivars. *Transactions of the ASABE* **2018**, *61*(3), 1165–1174. https://doi.org/10.13031/trans.12553
