# Supplementary material for: Proso Millet Cultivar Effects on Rheology of Dough and Quality Characteristics of Gluten-Free Breads
Source: Foods. 2026 May 13;15(10):1711. doi: 10.3390/foods15101711 (PMC13206684; doi:10.3390/foods15101711)
Supplement: Supplementary file 1 [file foods-15-01711-s001.zip › File S3_Cultivar bread pics.pdf]

Proso Millet Cultivar Effects on Rheology of Dough and Quality Characteristics of Gluten-Free Breads

Manjot Singh and Akinbode A. Adedeji *

Figure S1 (word format): Photographs of gluten-free breads prepared from nine proso millet cultivars

Bread cross-section

Sunrise	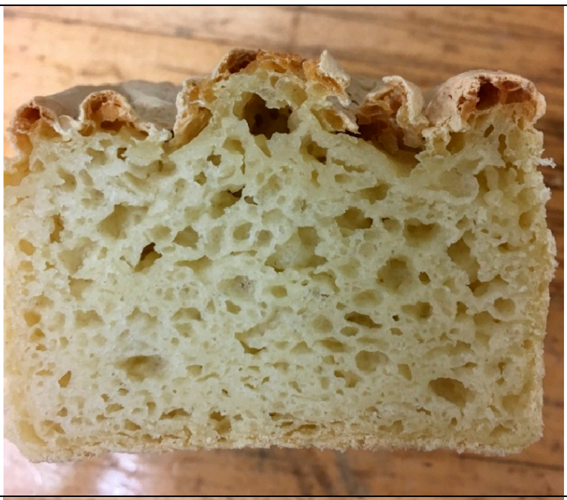	
Huntsman	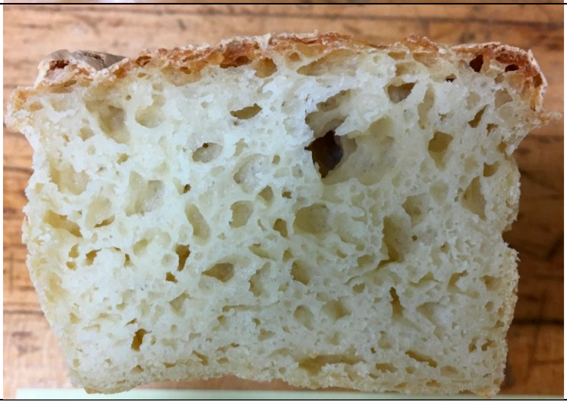	

Cope	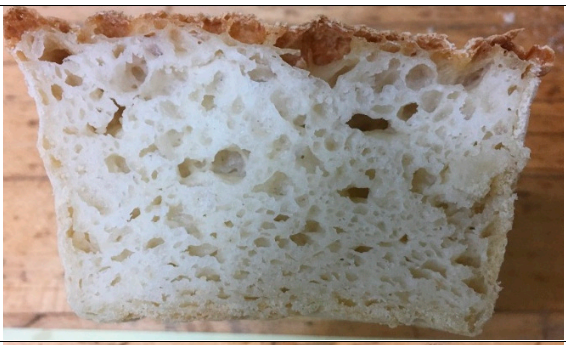	
Dawn	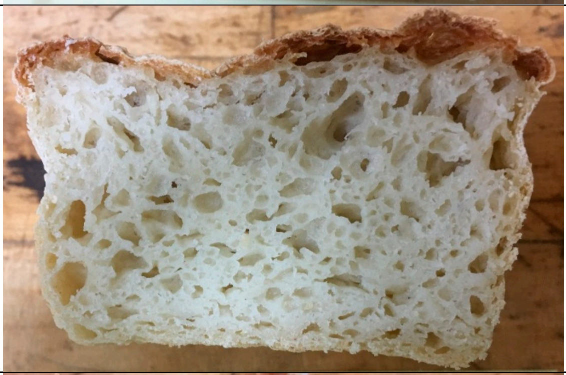	
Panhandle	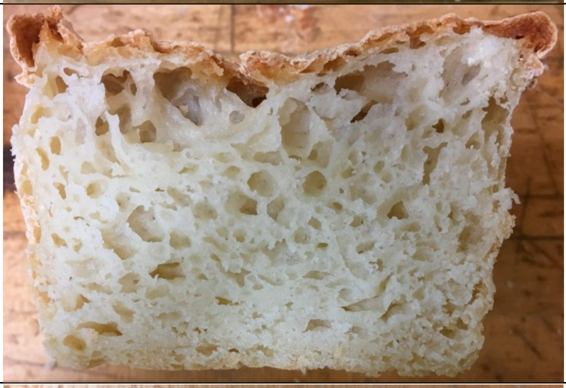	
Earlybird	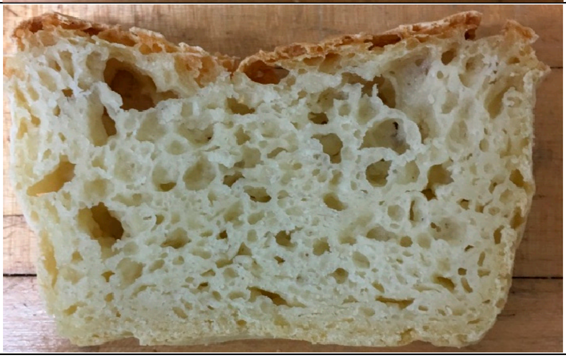	

Minco	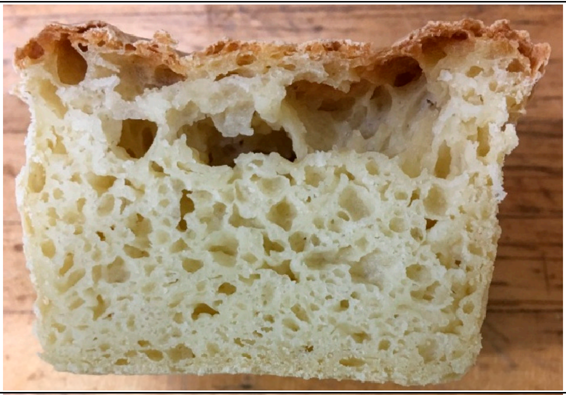	
Plateau	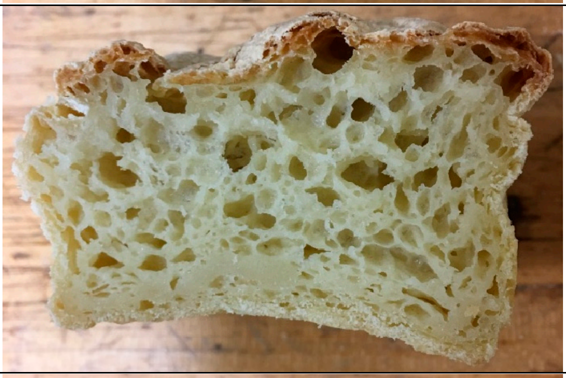	
Rise	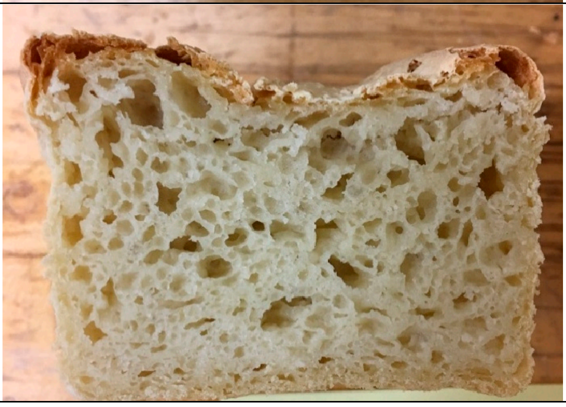	

Bread Side-view

Sunrise	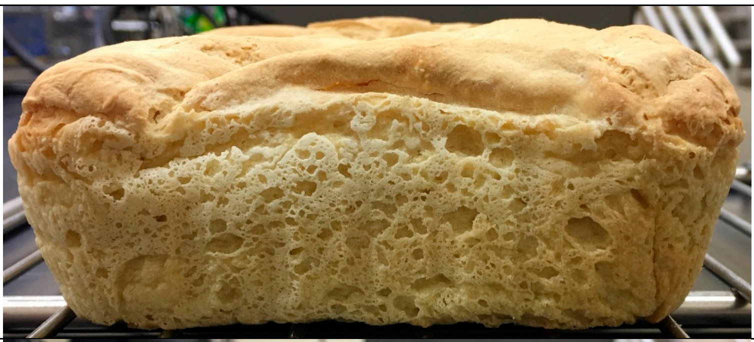	
Huntsman	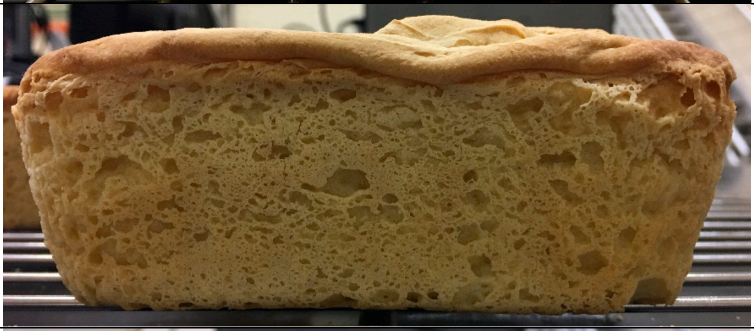	
Cope	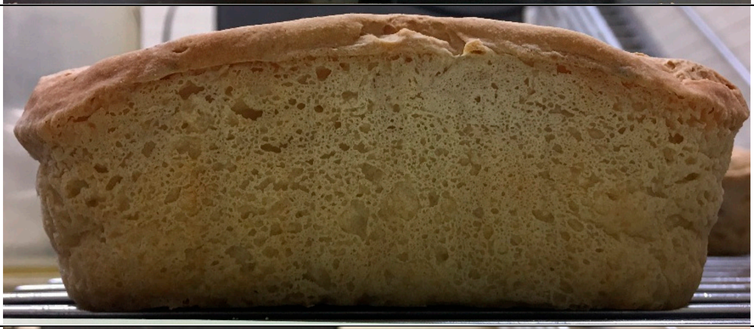	
Dawn	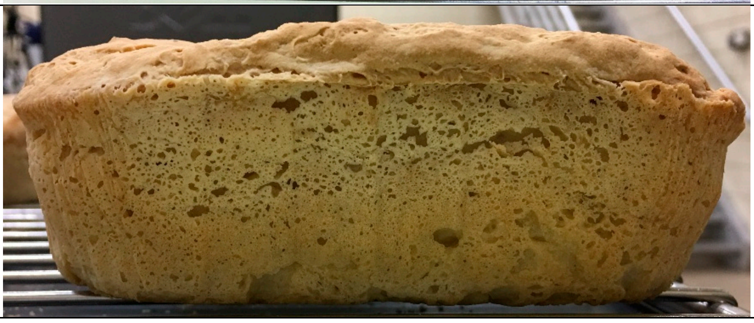	

Panhandle	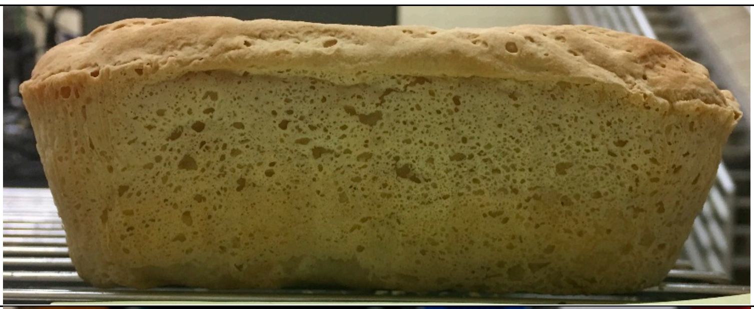	
Earlybird	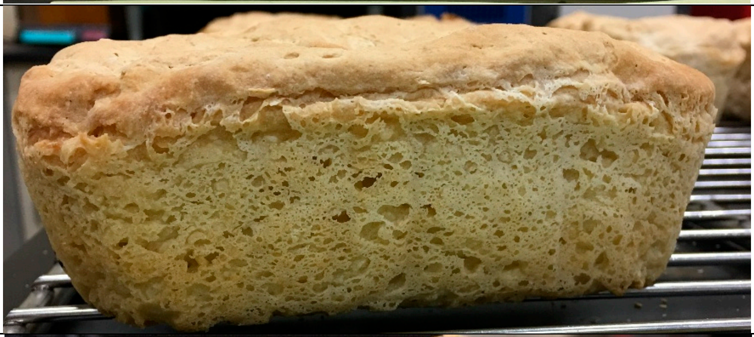	
Minco	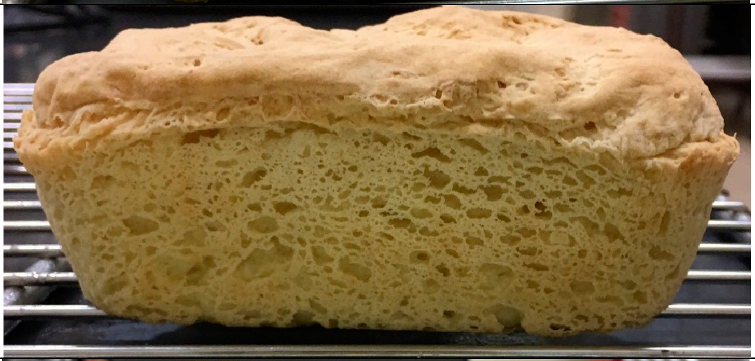	
Plateau	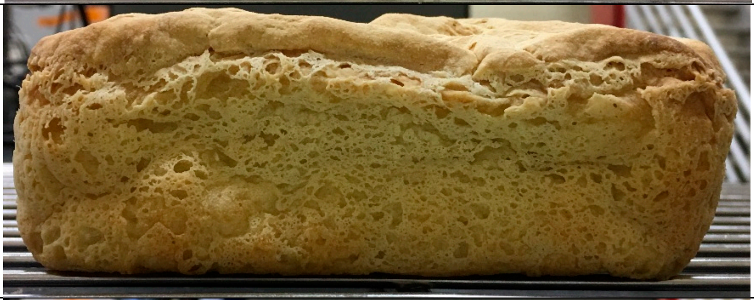	
Rise	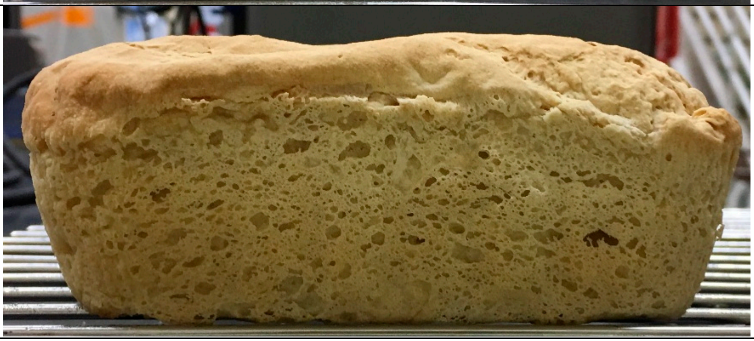	

Bread Top-view

Sunrise	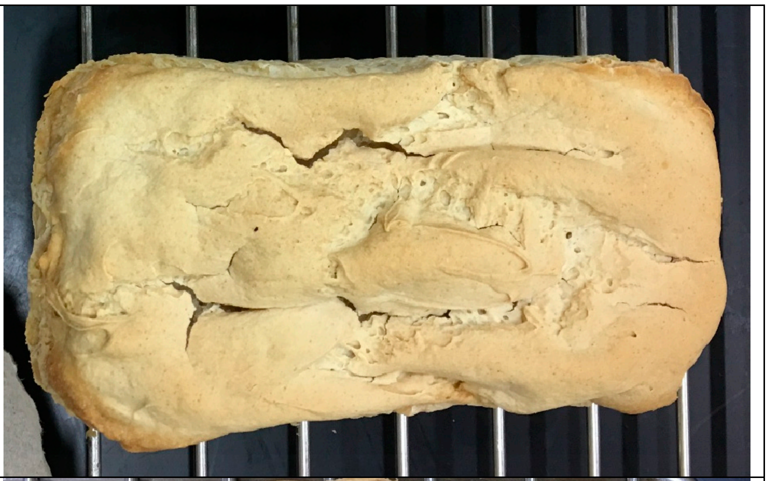
Huntsman	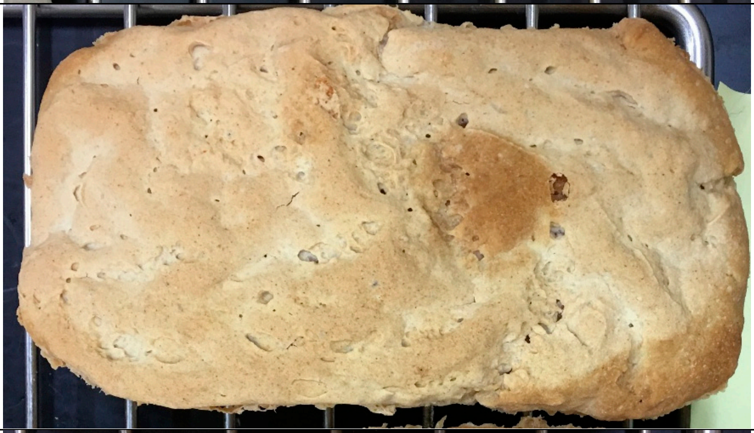
Cope	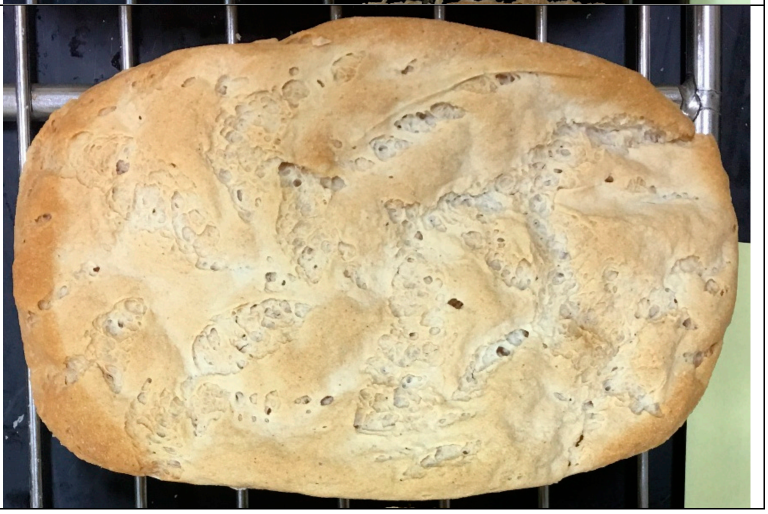

Dawn	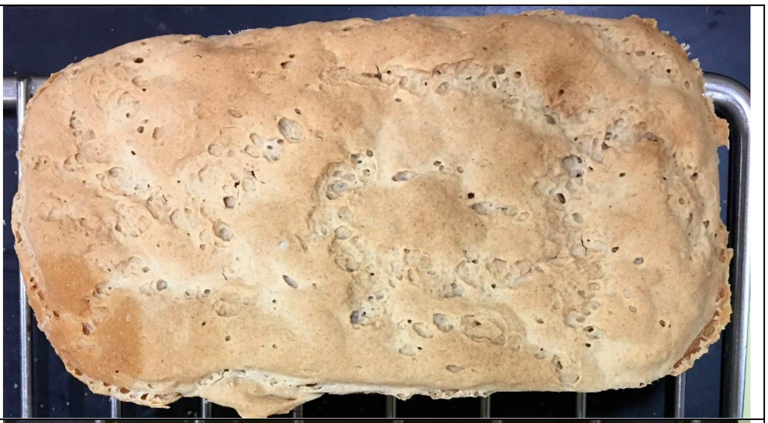
Panhandle	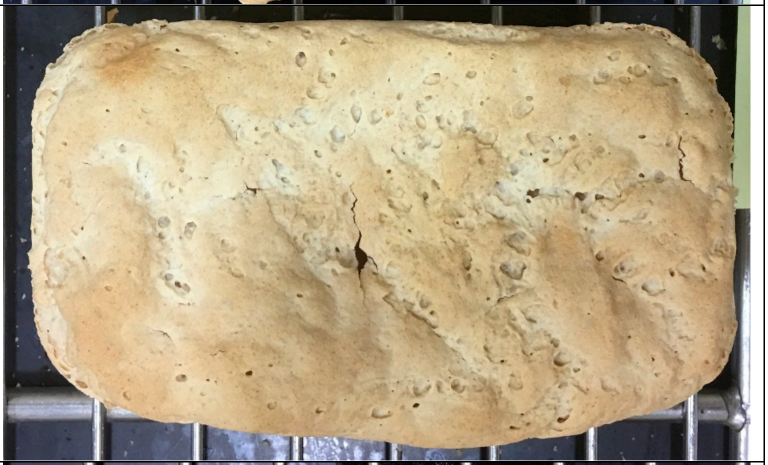
Earlybird	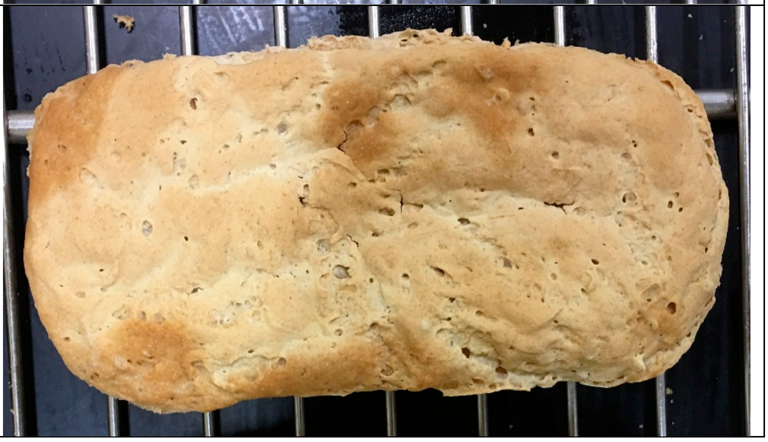

Minco	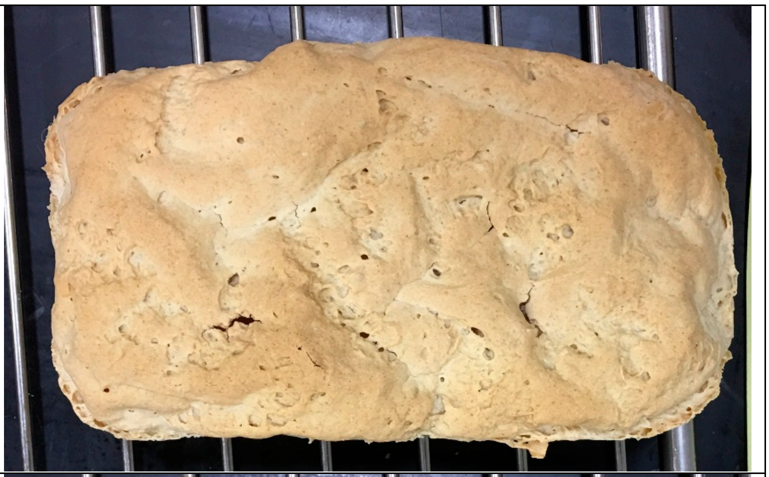
Plateau	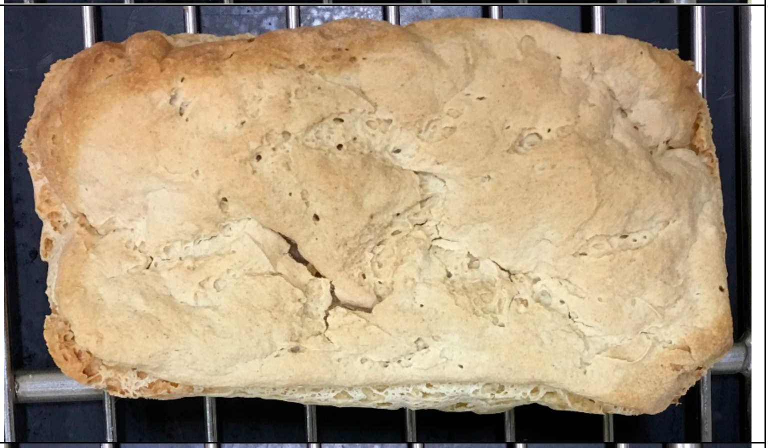
Rise	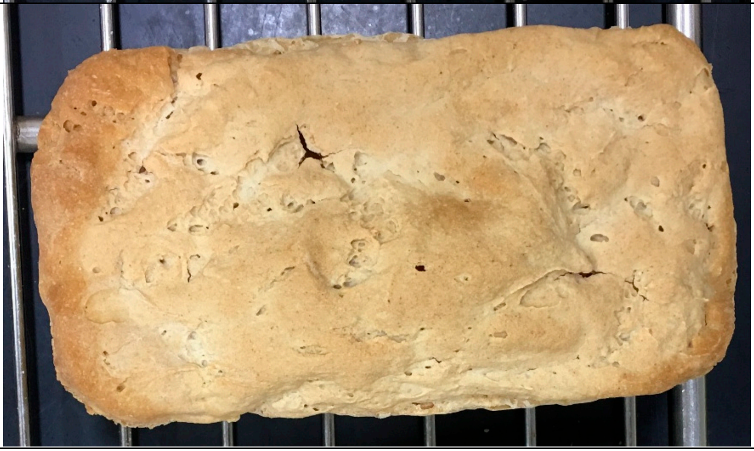
